# Immunohistochemical expression of estrogen receptor alpha in the maxillary sinus, pulp, and periodontal ligament of adjacent teeth in late pregnancy in rats

**DOI:** 10.1007/s10266-022-00770-0

**Published:** 2022-11-25

**Authors:** Gihan S. Hassan, Mai B. Helal, H. F. Ibrahim

**Affiliations:** Faculty of Dentistry, Tanta University, El-Giesh St., Tanta, Gharbia Egypt

**Keywords:** Immunohistochemistry, Estrogen receptor, Maxillary sinus, Dental pulp, Periodontal ligament

## Abstract

This study aimed to assess the histological changes in the maxillary sinus and its adjacent dental tissues as pulp and periodontal ligament during pregnancy and investigate the role of estrogen hormone in these changes through the detection of estrogen receptors in these tissues. Sixteen adult female rats were used and were allocated into two groups: control non-pregnant (*n* = 8) and pregnant (*n* = 8). They were sacrificed and their heads were prepared for histological and immunohistochemical examination for estrogen receptor alpha. Our results revealed that pregnant rats revealed inflammatory changes in the sinus as thick epithelial lining, loss of cilia, swollen goblet cells, intraepithelial and interstitial edema. The lamina propria demonstrated considerable infiltration of inflammatory cells, glandular hyperplasia with vacuolar degeneration, and vascular congestion. Periodontal ligament and pulp revealed hyperemia and vascular congestion. Immunohistochemical examination of estrogen receptor alpha in the maxillary sinus and adjacent dental tissues (Periodontal ligament and pulp) in pregnant rats revealed a significant increase in its expression in all examined tissues. In conclusion, there was an increase in expression of ERα in the sinus mucosa and dental tissues during pregnancy together with slight inflammatory changes in these tissues. Hence, dentists should be aware of the effect of these changes on the pregnant women avoiding teeth extraction due to misdiagnosis of dental, periodontal or sinus pain after exclusion of true pathologies.

**Supplementary Information:**

The online version contains supplementary material available at 10.1007/s10266-022-00770-0.

## Introduction

Pregnancy induces physical and hormonal changes that could adversely affect oral health [1]. Clinical investigations showed that between 38.2 and 54% of pregnant women suffered from dental pain [2]. This pain originates basically from caries, pulpal or periodontal involvement. Also, it may refer to teeth from the affected sinuses [3]. Nearly, 30% of pregnant women have experienced hypersensitivity to cold stimuli in maxillary teeth with tenderness to percussion provoking these patients to seek for dental care during pregnancy. However, the main cause of such dental complain was rhinosinusitis [4, 5].

Pregnancy rhinosinusitis is a widespread problem during pregnancy that involves inflammation of mucous membranes lining the nose and the maxillary sinus. Pregnancy rhinosinusitis is defined as nasal congestion, rhinorrhea and sneezing that emanate specifically during the last 6 or more weeks of gestation and usually dissolve completely 2 weeks postpartum excluding previous historyof allergic or pathological conditions. It is considered a serious condition causing persistent nasal congestion that acts as a potential risk factor in disturbing fetal growth and development through gradual hypoxia process [6].

Although the exact pathophysiology of pregnancy-associated alterations in the maxillary sinus and its adjacent dental tissues as pulp and periodontal ligament (PDL) is still not determined, hormonal changes during pregnancy play undeniable role. Interestingly, Robinson et al. [7] owed the raised frequency of oral diseases as periodontal diseases in pregnant women to changes in the levels of estrogen. Dental pulp, as PDL is composed of connective tissue; thus, it may also be affected by hormonal changes [8].

Estrogen hormone is a steroidal hormone that exerts profound effects mainly on the reproductive systems. Noteworthy, previous studies reported the impact of estrogen on oral and dental tissues. It was reported that estrogen deficiency altered the gene expression involved in craniofacial growth sites and odontogenic area leading to alterations in the jaws bone length, condylar growth and morphological deterioration of tooth structure, respectively [9–11]. These effects are mediated by estrogen receptors (ERs) that are one of the ligand-activated transcription factors which control the cellular differentiation and growth. There are two types of ERs: estrogen receptor alpha (ERα) and estrogen receptor beta (ERß). There is a noticeable tissue-specific difference in the distribution of ERs subtypes, with classic estrogen-target tissues expressing primarily the ERα subtype such as mammary glands and the endometrium [12].

Although medical literature provides studies regarding the high prevalence of pregnancy induced rhinitis and the associated changes of nasal cavity, scarce studies examined the potential histological changes in the maxillary sinus and its adjacent dental tissues (pulp and PDL) during pregnancy [13]. As the great resemblance between rodents and humans in the lining mucosa of the maxillary sinus and dental tissues, rats were used in this study. This study was designed to examine the histological changes and investigate the role of estrogen hormone in these changes through the detection of immunohistochemical expression of estrogen receptors in these tissues. Therefore, we hypothesized that increased estrogen during pregnancy caused inflammatory changes in the maxillary sinus and the adjacent dental tissues that may cause dental pain or discomfort.

## Materials and methods

In the current study, sixteen adult female Albino rats, 6–7 weeks of age, with an average body weight of 200–250 g, were purchased from pharmacology department, faculty of pharmacy, in Tanta University. They were kept for 1 week for acclimatization in the standard conditioned animal houses under controlled temperature, a 12-h alternating light/dark cycle. They were fed standard diet and tap water throughout the experimental period. Rats were randomly divided into two equal groups: non-pregnant (control) (*n* = 8) and pregnant (*n* = 8) rats. Sample size calculation was performed with G Power (version 3.1.9.4, Germany). Based on a previous study of Ciulla et al. [14], it was considered a means of 101.14 and 15.57 and a difference between standard deviation of 11.35, *α* = 0.05, and a power of 0.8. We have calculated a sample size of 7.5. Considering this result, we have used eight rats per group in this study. Also, a similar sample size was used in a former study [15]. This study was approved by the Ethical Committee of Faculty of Dentistry, Tanta university and were conducted in accordance with the guidelines laid down by the ARRIVE (Animal Research: Reporting In Vivo Experiments) guidelines for conducting animal research. Eight rats (pregnant group) were allowed to mate with males. The first gestational day was the day of formation of the vaginal plug.^1^

### Euthanasia and samples processing

**Fig. 1:**
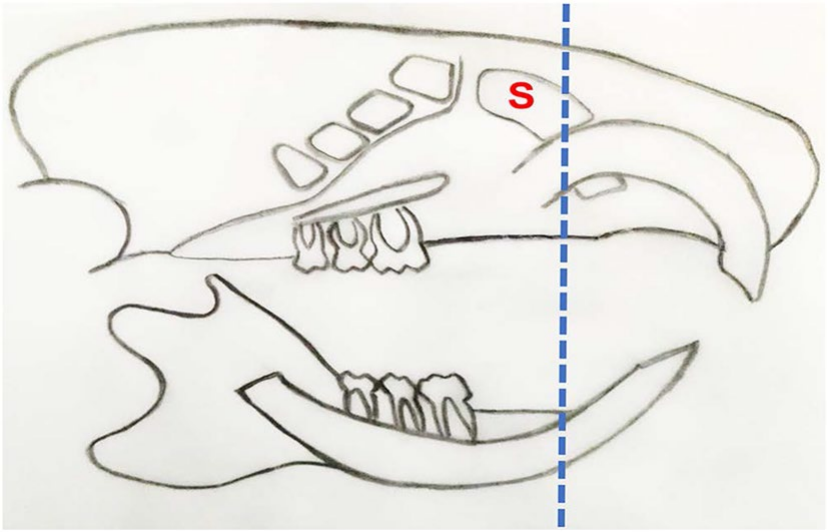
Schematic diagram of the area and the direction of cutting (dotted line). It illuminates the anatomical relation of the maxillary sinus (S) with maxillary incisors

Control rats and pregnant rats (on the 20th day of pregnancy) were anesthetized with ketamine chloride (Ketalar, 40 mg/kg body weight), Ketalar (par pharmaceutical companies, Inc. suffern, NY, USA). Then, they were euthanized by cervical dislocation to minimize pain and discomfort. The 20th day of pregnancy was selected as it was reported that the estradiol values were generally low except at the late pregnancy which is characterized by the maximum level of estrogen [16]. The heads of rats were dissected and cut coronally in front of their eyes where the maxillary sinuses are located as shown in the diagram (Fig. 1). Then, specimens were immediately fixed in buffered formaldehyde (pH: 7.4) for 48 h.

### Histological and immunohistochemical examination

Histological examination was done for the maxillary sinus and adjacent dental structures. These dental structures were represented by dental pulp of the rat incisor and its surrounding PDL. They were decalcified in 10% EDTA for 4 weeks. They were washed in tap water over night and then dehydrated in ascending grades of alcohol, cleared in xylol, and embedded in paraffin wax (56–58 °C melting point). Sections of (5 μm) were cut using a rotary microtome (leica) and then stained with hematoxylin and eosin for light microscopic examination (LM).

#### Immunohistochemical examination

Avidin-Biotin-Complex (ABC) method was used for immunohistochemical labeling to investigate ERα expression within the maxillary sinus and its closely associated dental structures via procedures previously reported [17, 18]. Briefly, deparaffinized sections were initially incubated with 30% volume of 3% hydrogen peroxide and 70% volume of absolute alcohol. The slides were then washed in running water for 3 min. The specimens were preheated at 100 °C for 10 min (for antigen retrieval) and incubated in H_2_O_2_ (for inhibition of endogenous peroxidase). After autoclave treatment (5 min at 121 °C in citrate buffer), the sections were incubated in blocking solution (10% normal goat serum in PBS) for 30 min before incubation in primary antibody (clone 1D5, Dako, Carpinteria, CA, USA). The primary antibody anti ERα (Mouse monoclonal [1D5] to ERα) was applied at dilution of 1:50 and incubated at 4 °C overnight. Sections were carefully washed three times with phosphate buffered saline (PBS) for 15 min and incubated with the secondary antibody, a peroxidase conjugated polyclonal goat anti-mouse antibody (Becton Dickinson, Milan, Italy) at a dilution of 1:250 for 1 h at room temperature. Thereafter the tissue sections were incubated for 30 min at room temperature with a horseradish peroxidase-avidin biotin complex (Vectastain Elite, Vector, CA). The site of bound enzyme was visualized by the application of 3,3-diaminobezidine in hydrogen peroxide (DAB kit, Vector, CA), a chromogen which produce a brown, insoluble precipitate when incubated with the enzyme. Afterwards, the background was counterstained with Mayer’s hematoxylin. Sections were gradually dehydrated and mounted with coverslips and examined under light microscope by blinded examiners. Slides stained with secondary antibody only were used as negative controls and breast tissue specimens were used as positive control for ERα.

ERs expressing cells contained brown cytoplasmic labeling. The intensity of immunostaining measurement was performed on the images using the Image J analysis software (Image J 1.42q, Wayne Rasband, USA). To quantify the positive immune reaction, we followed that described in previous study [19]; Quantitative morphometric measurements were carried out blindly using 25 observations. This was performed in 5 non-overlapping fields from 5 different sections for each specimen of total 16 specimens for both control and pregnant groups at magnifications ×400. These different fields were examined for the mean intensity gray value. Then, the optical density (OD) values were identified by using this formula OD = log (maximum intensity/mean intensity), where max intensity = 255 for 8-bit image.

### Statistical analysis

The quantitative numerical data that were collected from the measurement of the intensity of estrogen immunostaining, were tabulated and presented as mean ± standard deviation (SD) with taking possibility of hypothesis H0 two tailed. Normality of data was checked using the Shapiro–Wilk test. Comparison of the mean difference of intensity regrading PDL, pulp and the maxillary sinus between the groups was done by unpaired *t* test. The significance level was set as *P* value ≤ 0.05 is significant. Statistical analysis was performed using statistical package for social sciences (SPSS) version 16.

## Results

### Effect of pregnancy on the histology of the maxillary sinus and the adjacent dental structures

**Fig. 2:**
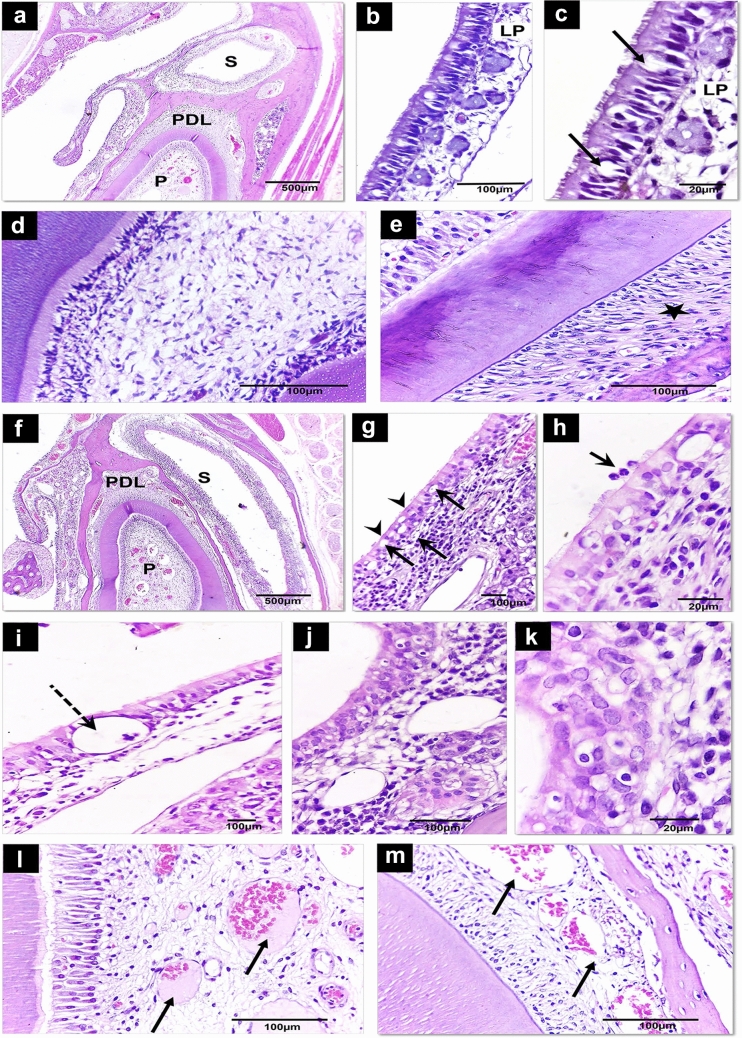
**a**–**e** Light micrograph of control (non-pregnant) rats sinus, pulp and PDL. **a** Showing the anatomical relation between maxillary sinus, pulp and PDL. **b** It reveals normal thin maxillary sinus lining and underlying lamina propria (LP). **c** Higher magnification showing normal pseudostratified, ciliated, columnar epithelium with goblet cells (arrows) lines the maxillary sinus. The lamina propria (LP) contains small islands of seromucous glands. **d** The dental pulp tissue displays normally arranged pulp tissue. **e** normal PDL that shows well-organized oblique fibers (star). **f**–**m** Light micrographs of the sinus, pulp and PDL of pregnant rats. **f** It displays changes within maxillary sinus, pulp and PDL of pregnant rats. **g** The sinus lining reveals loss of cilia from some areas of the columnar epithelial cells (arrowheads), intraepithelial and interstitial edema (arrows), subepithelial inflammatory cell infiltrate are present. Notice, vascular congestion, and glandular hyperplasia in the underlying lamina propria. **h** The sinus lining displays penetration of few inflammatory cells (arrow) into the sinus lumen. **i** Intraepithelial cyst (dashed arrow) and interstitial edema. **j** The epithelial lining reveals squamous metaplasia. Also, the lamina propria shows inflammatory cell infiltrate. Notice, dilated capillaries in the underlying lamina propria together with glandular hyperplasia. **k** Higher magnification displays epithelial squamous metaplasia and heavy subepithelial infiltration of inflammatory cells. **l** The dental pulp core reveals vascular hyperemia and congestion (arrows). **m** The PDL exhibits vascular congestion (arrows). *P* pulp, *S* sinus, *PDL* periodontal ligament. (Hematoxylin and eosin [H&E], scale bar: 500 µm (**a**, **f**); 100 µm (**b**, **d**, **e**, **g**, **i**, **j**, **l**, **m**); 20 µm (**c**, **h**, **k**)

This study examined the histological changes of the maxillary sinus and adjacent dental tissues in non-pregnant (control) (Fig. 2a–e) and pregnant rats (Fig. 2f–m). The histological features of the maxillary sinus of the non-pregnant control group displayed a thin layer of normal pseudostratified ciliated columnar epithelial lining with goblet cells. Immediately beneath the epithelium, it showed a thin uniform zone of loose connective tissue, the lamina propria. Small islands of seromucous glands along with blood capillaries were dispersed throughout lamina propria. Basement membrane separated the epithelial layer from the lamina propria (Fig. 2a–c). On the other hand, sinus mucosa of the pregnant rats showed histological features suggesting sinusitis. The epithelial lining was thick and revealed loss of cilia from some areas of columnar epithelial cells (Fig. 2g, h). Within the epithelium, several goblet cells appeared swollen and distended with mucous. Also, there was intraepithelial and interstitial edema (Fig. 2i). The lamina propria demonstrated considerable infiltration of inflammatory cells. Although the inflammatory cells were mostly accumulated in the superficial layer of the lamina propria, few inflammatory cells showed penetration into the epithelial layer (Fig. 2h). Also, glandular hyperplasia with vacuolar degeneration and vascular congestion in lamina propria were detected (Fig. 2j, k).

Regarding the histological features of the pulp and PDL of the adjacent teeth, the control group displayed normal histological features. The dental pulp revealed normally arranged pulp zones (Fig. 2d). Also, PDL fibers were well organized (Fig. 2e). However, the dental pulp and PDL in the pregnant rats revealed inflammatory changes in the form of vascular congestion and hyperemia (Fig. 2l, m).

### Effect of pregnancy on the intensity of ERα expression

**Fig. 3:**
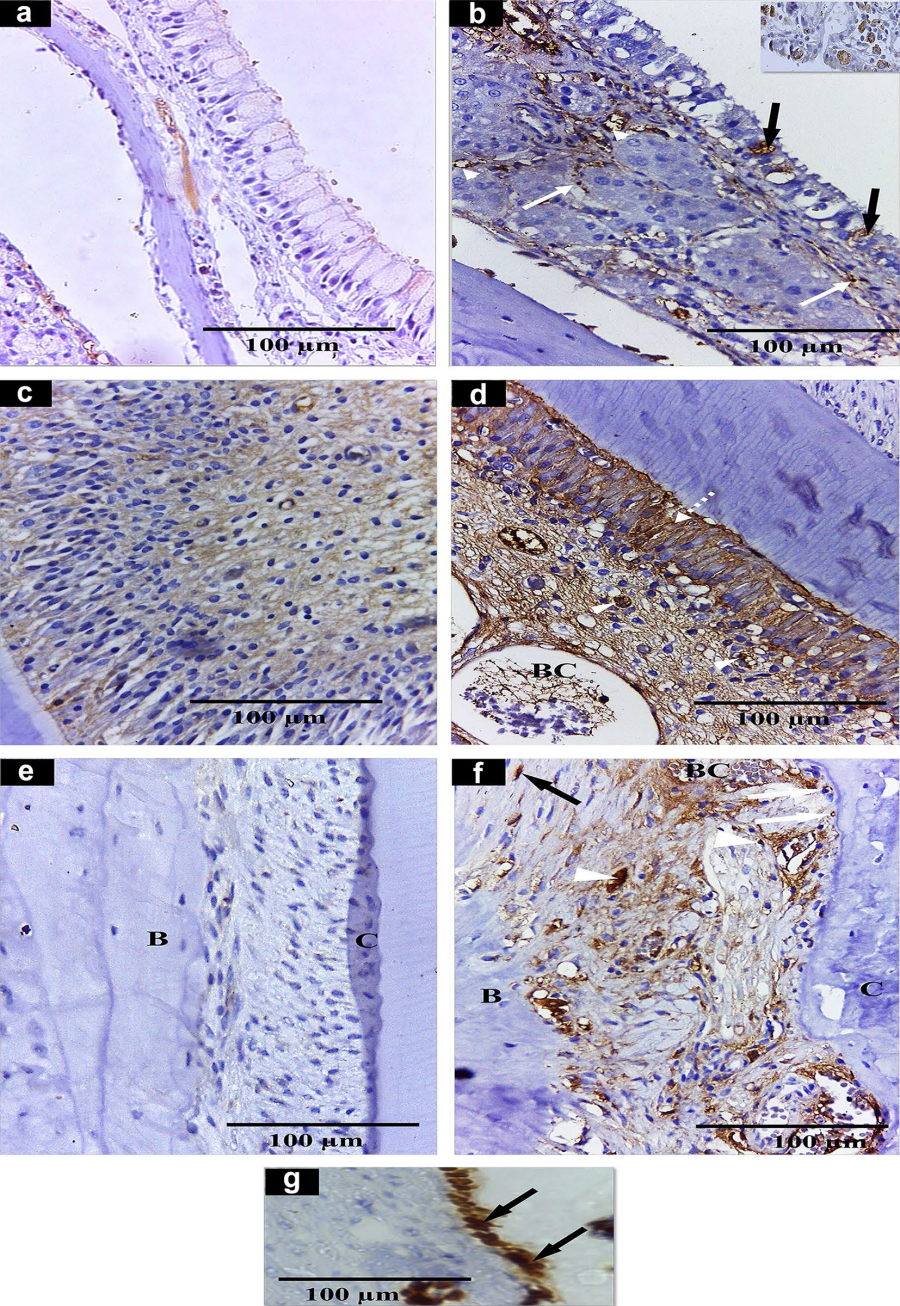
**a**, **b** Light micrographs of maxillary sinus submitted to immunohistochemical staining for detection of ERα expression. **a** The control group shows negative reaction in epithelial cells. **b** Pregnant rats showing positive expression of ERα within endothelial cells lining blood capillaries walls (arrowheads), cells of the lamina propria (white arrows), as well as sporadic staining among goblet cells (black arrows) within the epithelial lining. Inset shows a strong reaction of mast cells. **c** The dental pulp of the control group shows mild cytoplasmic expression in the stromal cells. **d** The pulp of pregnant rats reveals strong cytoplasmic staining in odontoblasts (dashed white arrow), stromal cells (white arrowheads), and endothelial cells lining of blood capillaries (BC). **e** PDL of the control group shows negative reaction. **f** PDL in pregnant rats, depicts strong immunostaining in fibroblast (black arrow), stromal cells (white arrowheads), cementoblasts (white arrows), and endothelial cells lining of blood capillaries (BC), *B* bone, *C* cementum **g** immunohistochemical staining for detection of ERα expression in the breast as a positive control (arrowheads). (ERα immunostaining counterstain with Mayer’s hematoxylin. Scale bar:100 µm (**a**–**g**)

In control group, PDL and maxillary sinus displayed negative immunohistochemical reaction for ERα in epithelial cell lining, lamina propria and endothelial cells (Fig. 3a). Also, pulp cells exhibited a negative immunohistochemical reaction for ERα (Fig. 3c, e). Whereas immunohistochemical localization for ERα in pregnant rats depicted positive immunoreactivity in the maxillary sinus lining, PDL and pulp (Fig. 3b, d, f) compared to control group. Figure 3g displays immunohistochemical reaction for ERα in breast tissue as a positive control for ERα.

### Statistical analysis

The mean values regarding intensity level of ERα of control group were 13.58 ± 2.892, 23.63 ± 2.015 and 33.60 ± 7.508 for PDL, pulp, and maxillary sinus, respectively, whereas the mean values regarding the intensity level of ERα of pregnant group were 77.25 ± 2.673, 62.33 ± 6.493 and 123.7 ± 21.81 for PDL, pulp and maxillary sinus, respectively. In pregnant rats, upon using unpaired *t* test, the expression levels of ERα showed statistically highly significant increase (*P* value < 0.001) in the maxillary sinus, PDL and pulp tissues compared to control group. Quantitatively, ERα immunostaining intensity revealed a significant increase in the maxillary sinus and the adjacent dental tissues (pulp and PDL) in pregnant rats due to raised immunostaining intensity as shown in (Table. 1).

**Table 1:** Inter-group comparison of the mean values regarding the intensity of the periodontal ligament, pulp, and the maxillary sinus, respectively

Intensity	PDL^∞^		Pulp		Sinus	
	Control	Pregnant	Control	Pregnant	Control	Pregnant
Mean ± SD	13.58 ± 2.892	77.25 ± 2.673	23.63 ± 2.015	62.33 ± 6.493	33.60 ± 7.508	123.7 ± 21.81
*t* test	45.73		16.10		11.05	
*P* value	< 0.001***		< 0.001***		< 0.001***	

Unpaired *t* test significance: p* < 0.05, p** < 0.01, p*** < 0.001

## Discussion

Pregnancy initiates unique changes in women’s bodies which markedly affect their health [20]. Clinical studies assumed a connection between the pregnancy hormones and the nasal mucosa [18, 21]. Also, previous studies reported increased frequency of gingival diseases in women during certain life periods as pregnancy and during contraceptives usage [22, 23]. However, scarce data are available on the effect of pregnancy on the sinus, PDL and dental pulp, hence, this study was mandatory to illustrate the relation between the pregnancy and the histological changes in these tissues.

The female rats were used due to the greater similarity shared between rodents and humans in the lining mucosa of the maxillary sinus [24] and rats develop sinusitis only by induction through administration of antigenic substances, or specific bacteria [25] thus the existence of sinusitis is mainly due to elevated estrogen. Also, Hassan et al. [26] declared that elevated sex hormones during pregnancy increased the proliferative activity of gingival epithelium as rats lack the initiating factors for gingivitis such as plaque and calculus. Therefore, using rats excludes predisposing factors that might potentiate the effect of pregnancy hormones on various tissues. Noteworthy, incisors were selected in our study though their different continuous eruption pattern because of their close anatomical relationship to maxillary sinus in rats. Phillips et al. [27] studied the anatomy and volume of the paranasal sinus cavities from two-dimensional (2-D) and three-dimensional (3-D) CT images. They described the anterior maxillary sinus of rat to be located medial to the caudal end of the upper incisor tooth root while the posterior maxillary sinus is located superior to the caudal end of the upper incisor tooth root.

In the current study, the control group disclosed the normal lining of the maxillary sinus. This was in agreement with Edranov et al. [24] in Albino rats. On the other hand, the lining of the maxillary sinus of pregnant rats displayed various signs of inflammation, which were presented in the form of vascular congestion, glandular hyperplasia, swollen goblet cells, squamous metaplasia, intraepithelial oedema, perivascular oedema, intraepithelial cysts, pseudo-cysts, basement membrane thickening and loss of cilia. Such inflammatory changes coincided with Poerbonegoro [28] who presented a series of observations including signs of rhinitis and increased volume of the inferior turbinate during menstruation and sexual stimulation. Also, Toppozada et al. [29] found similar findings in respiratory nasal mucosa in women taking contraceptive drugs containing estrogen.

The inflammatory changes reported in the sinus mucosa of pregnant rats could be attributed to the breakdown of the extracellular matrix by proteases matrix metalloproteinase (MMP-9) leading to pseudo-cysts. Thickening of the basement membrane was due to the accumulation of fibronectin and types I, III and V collagens [30]. The later changes were consistent with Güven and Ortuǧ [15], who reported a change in intracellular actin cytoskeleton dynamics in response to fluctuation in estrogen hormone levels.

In our study, both dental pulp of pregnant rat incisor and its surrounding PDL disclosed similar histological changes represented by vascular congestion and hyperemia emphasizing the effect of pregnancy on dental tissues though the different continuous eruption pattern of rat incisors. This agreed with Dzeletovic et al. who reported an increase in pulp blood flow during the menstrual cycle [31]. Also, this finding were in accordance with Nebel et al. who declared increased vascular permeability of PDL during pregnancy [32]. Noteworthy, the histological changes of dental pulp were mimicking the histological features of reversible pulpitis [33] suggesting that these structural modulation could be a transient effect possibly caused by pregnancy hormonal factors.

Pregnancy has been proved to cause prominent fluctuation in estrogen and progesterone levels by 30 and 10 times, respectively [34]. As well, estrogen action on various tissues is mediated through its receptors [35]. Hence, it was essential to detect changes in expression of receptor ERs during pregnancy.

Our immunohistochemical findings suggested site-specific effect of estrogen hormone during pregnancy. This was evidenced by the significant increase in expression of ERα within the maxillary sinus, dental pulp and PDL of pregnant rats compared to their comparable parts of the control rats. Consistent with our results, Zhao et al. [21] detected a marked increase in ER expression levels in nasal polyps in human upper airways. They attributed the effects of estrogen on airways to this increase in estrogen receptors which represented the principal target for its action. Also, Mata et al. [36] found an increase in ER expression in endothelial and vascular smooth muscle layers in pregnant rats. Also, Shu et al. [37] explored that estrogen, acting through ERα and ERβ located in PDL cells, may have a significant impact on the periodontium. It has the ability to modulate bone-resorbing cytokines such as interleukin 6 and 1 beta, tumor necrosis factor alpha, receptor activator of nuclear factor kappa-B ligand as well as osteoprotegerin in PDL cells.

The change in the expression of ERα reported in our immunohistochemical results suggested a relation between estrogen hormone and the histological changes of the examined tissues. This coincided with Ribeiro-Dasilva et al. [38], who studied ERα expression in the temporomandibular joint. They declared that amplified inflammation was related to augmented ERα expression. They also showed the pro-inflammatory effect of ERα on monocytes. Noteworthy, temporomandibular disorders in the form of disc displacement and arthralgia have been linked to genetic polymorphisms in *ESR2*, which are genes encoding estrogen receptor [39].

The mechanism of action of estrogen could be explained by the anticholinergic activity of estrogen hormone that could explain both the vascular changes and the glandular hyperplasia within the affected tissues of pregnant rats [40, 41]. Also, estrogen hormone could mediate an allergic effect as a form of hormonal allergy that could trigger an immune reaction within the affected tissues. This explanation agreed with Bonds and Midoro-Horiuti [42] who hypothesized that ER, progesterone, as well as their metabolites may play a role as antigens after they bind to different proteins, boosting T helper 2 (Th2) cell development and the synthesis of antibodies. These antibodies bind to mast cells with their corresponding antigens (hormones or metabolites) leading to their degranulation and release of histamine, leukotriene, and Th2 cytokine causing Type I allergy. Also, they revealed that mast cell degranulation led to rapid oedema of smooth muscles and recruitment of other inflammatory cells to the inflammatory site. Similarly, Bingham and Austen [43] declared that estrogens might support allergic responses via ERα on mast cells which explain the highest allergic reactions in females. Moreover, estrogen could mediate an allergic effect by increasing histamine receptors in the epithelial cells and the associated microvasculature [44].

Moreover, estrogen could have a regulatory role on inflammatory process. It has been reported that estrogen can induce inflammation through the stimulation of toll-like receptors (TLR) through signaling monocytes, macrophages, and immune cells in the central nervous system. These cells release pro-inflammatory cytokines that initiate inflammation and contribute to its progression [45]. In addition, estrogen diminishes the immunity, enhancing the affinity and severity of oral inflammation [46].

Noteworthy, Filippini et al. [47] disclosed that TLR central activation may participate in dental nociceptive pain transmission in pulp. Bi et al. correlated temporomandibular joint-related pain to the change in estrogen levels. They found that estrogen could mediates inflammation, through the upregulation of voltage-gated sodium channels enhancing nociception and hyperalgesia [48]. Ribeiro-Dasilva et al. [49] declared that estrogen receptors are present in the dorsal root ganglion and trigeminal nerve nucleus, therefore, the oscillations of estrogen level may change the opioid systems function, boosting perception of pain. Keita-Alassane et al., reported that woman’s pain threshold may be influenced by instability of estrogen levels [50]. Fortunately, dental pain does not occur in all pregnant women. This may be explained by Toppozada et al. [29] who correlated this to the selective estrogen hypersensitivity or to variations in the sex hormones production.

Finally, a few potential limitations need to be considered. First, our study has focused on immunohistochemical expression of only one estrogen receptors together with lack of gene expression analysis in the research area. Second, the present study has only investigated one period, late pregnancy. Consequently, situation is thus still incomplete. Future studies on the current topic are, therefore, required.

In conclusion, our results suggest increased expression of ERα in the sinus mucosa and dental tissues during pregnancy together with slight inflammatory changes in these tissues. The hypothesis that during pregnancy inflammatory changes are induced within the maxillary sinus and adjacent dental tissues caused by increased expression of estrogen receptors was accepted. Therefore, it is recommended that dentists be aware of the effect of these changes on the pregnant women avoiding teeth extraction due to misdiagnosis of dental, periodontal or sinus pain after exclusion of true pathologies. Also, further studies should be done on how to prevent this pain in pregnant women.

## Supplementary Information

Below is the link to the electronic supplementary material.

Supplementary file1 (PDF 154 KB)

## Data Availability

All data of this study are available from the corresponding author upon request.

## Footnotes

1 The normal gestation period for Albino rats is 21 days.

## Declarations

### ARRIVE guidelines

This study has been carried out according to the ARRIVE guidelines for the reporting of in-vivo experiments in animal research. The ARRIVE checklist can be found in the supplementary information.
